# Dr. Drake’s Work

**Published:** 1854-12

**Authors:** 


### DR. DRAKE'S WORK.

The second volume of Dr. Drake's great work on the *Diseases of the Interior Valley of North America* has been issued from the press of Lippincott, Grambo & Co., and is for sale, in this city, at Maxwell's bookstore. For the practising physician this volume will be found to exceed in interest the first, devoted as it is to practical medicine. No labors have reflected more honor upon the profession of our country than those of Dr. Drake, and these, the crowning ones of his life, we trust, will meet from the profession a fitting reception.

